# Distal Hamstring Injuries and Disorders

**DOI:** 10.5435/JAAOSGlobal-D-25-00248

**Published:** 2025-09-17

**Authors:** Dylan S. Koolmees, Jeffrey D. Klott, Tanner R. Poppe, David L. Bernholt

**Affiliations:** From the University of Missouri Orthopedic Surgery, Columbia, MO (Dr. Klott), and the Campbell Clinic Orthopaedics, University of Tennessee, Germantown, TN (Dr. Koolmees, Dr. Poppe). David L. Bernholt is now Department of Orthopaedic Surgery, University of Cincinnati.

## Abstract

Hamstring injuries are a common injury sustained by athletes with most injuries occurring as strain injuries within the muscle belly or at the proximal musculotendinous junction. Distal hamstring pathology is relatively uncommon but comprises a collection of both acute and chronic diagnoses that can manifest with symptoms either on the medial or lateral side of the knee based on which hamstring tendons are involved. Pes anserinus bursitis is the most common of these distal hamstring pathologies with other chronic diagnoses, including snapping medial hamstrings or snapping biceps femoris. Acute biceps femoris ruptures can occur in an isolated fashion but most often occur in the setting of concomitant posterolateral corner injury as a result of high-energy trauma. Isolated semitendinosus ruptures can occur with lower-energy acute events, commonly with track and field events. Most distal hamstring pathology can be treated without surgery and do well with conservative treatment. However, acute avulsion injuries often require surgical intervention, as can chronic problems that do not adequately respond to prolonged conservative treatment. Treatment algorithms for distal hamstring injuries are less well-developed than more proximal injuries owing to their lower incidence. This review focuses on distal hamstring injuries, the state of current literature, and treatment strategies.

Among athletes, the most commonly injured muscle group is the hamstring muscle group, comprising 12% to 29% of all sports-related injuries.^1–4^ Most hamstring injuries are noninsertional injuries sustained at the myotendinous junction as strain injuries, with the proximal musculotendinous junction being the most common location of injury. Proximal tendinous avulsions represent the next most commonly encountered area of injury. There is a large amount of literature discussing the diagnosis, workup, and treatment of both hamstring myotendinous junction/muscle strains and proximal avulsion injuries.^3^ Distal hamstring injuries occur less frequently, and at this time, there are only a limited number of case series and case reports discussing evaluation and treatment of distal hamstring pathology. For the sake of this review, we are defining distal hamstring pathology as problems that occur distal to the distal myotendinous junction of the hamstring tendons.

Broadly speaking, distal hamstring pathology can be categorized as traumatic or atraumatic lesions. Acute, traumatic injuries of the distal hamstrings most commonly involve the biceps femoris tendon and most often occur as part of a mult-ligamentous injury of the knee in which the posterolateral corner is involved.^1^ However, isolated biceps femoris avulsions do occur and have been reported in the setting of preexisting tendinopathy and in young athletes involved with running, jumping, kicking, and/or rapid acceleration in their sport.^5–8^ Distal hamstring avulsions can occur on the medial side as well, although less commonly than with the biceps femoris. These injuries most often occur as an avulsion of the semitendinosus with retraction proximal to the level of the knee joint, resulting in a painful mass.^8–10^ In addition, patients can present with “snapping” distal hamstring tendons that can be difficult for the treating physician to treat if they are not aware of the condition and may predispose the patient to wrong diagnoses or treatments.^11–14^ It is the intent of this literature review to discuss the hamstring anatomy and explore further the literature currently present on evaluation, diagnosis, and treatment of distal hamstring pathology to better educate the treating physician on these conditions.

## Anatomy, Mechanism of Injury, and Evaluation

The hamstrings are made up of three muscles: the biceps femoris, semitendinosus, and semimembranosus. The biceps femoris is unique in that it consists of two heads: the long and short heads. Except for the short head of the biceps, they all originate from the ischial tuberosity. This means that the long head of the biceps femoris, the semimembranosus, and the semitendinosus all span both the hip and knee joint; in addition, they are all innervated by the tibial portion of the sciatic nerve. The semimembranosus origin is more lateral on the ischial tuberosity, and in most patients, there is a common origin of the semitendinosus and biceps femoris that attaches more medially on the tuberosity. The short head of the biceps originates from the Linea aspera, is innervated by the common peroneal nerve, and inserts on the lateral proximal fibula and tibia, therefore only spanning the knee joint. The semitendinosus has the smallest cross-sectional area of the hamstring muscle group, whereas the semimembranosus is the largest.^15,16^

At the hamstring's distal insertion, the semimembranosus and semitendinosus attach on the medial tibia, whereas the biceps femoris' two heads joint together to attach on the lateral aspect of the knee at the proximal fibula and lateral tibia. These muscles can therefore act as horse reins around the knee joint to aid in rotational stability and reinforce the capsule while stabilizing the posterior structures such as menisci.^16^ The insertion of the semimembranosus is highly complex, and it divides into five branches at the level of the joint line that help it play a role in posteromedial stability of the knee, as it also intertwines with the oblique popliteus ligament; in addition, it also retracts the posterior horn of the medial meniscus during knee flexion, which keeps the meniscus from being crushed during deep knee flexion.^16–18^ The semitendinosus insertion is part of the pes anserine tendon complex, which includes the gracilis, semitendinosus, and sartorius. The semitendinosus inserts deep to the attachment of the sartorius and anterior/inferior to the gracilis in the pes anserine complex. The semitendinosus and gracilis tendon are commonly harvested tendons for ACL reconstruction. However, the semitendinosus can assist with knee flexion, tibial internal rotation, and dynamic stability,^19,20^ with some suggesting that it is important to knee flexion strength at increased flexion angles, such as for hurdlers and football defensive backs.^21,22^ Cadaveric anatomic studies have provided pertinent osseous landmarks of the distal biceps femoris four insertional footprints: a tibial footprint, proximal fibular footprint, distal fibular footprint, and medial fibular footprint.^23,24^ The proximal and distal fibular footprints are mostly supplied by the long head fibers, medial fibular footprint by the short head, and both heads contribute to the tibial head. ^24^ The attachment of the fibular collateral ligament to the fibular head lays in between the proximal and distal fibular footprints of the biceps femoris.^24^ The biceps femoris has a very broad insertion on the fibular head when considering its proximal, distal, and medial fibular attachments, which should be considered when planning surgical fixation of an avulsed biceps femoris tendon. The biceps femoris is involved in lateral stability of the knee through its tie into the posterolateral corner complex in combination with the popliteus muscle, popliteofibular ligament, fibular collateral ligament, and posterolateral articular capsule. It can act as a dynamic and static knee stabilizer, as well as, resisting tibial internal rotation torque.^25^

Injury to the hamstrings typically occurs with sudden hip flexion and knee extension. Hamstring injuries occur more commonly in rapid acceleration sports like track and field, football, and soccer. The most common area of injury to the tendon is at the myotendinous junction; however, avulsion injuries do occur. Diagnosis of hamstring injuries mainly relies on the physical examination; however, patients can report a “pop” in the back of their leg or describe an incident of someone kicking them in the back of the thigh. They will usually report pain, stiffness, and weakness. If there is clinical concern for a high-grade distal hamstring injury, diagnosis can be confirmed with imaging, with MRI being the most commonly chosen modality to evaluate tear pattern and retraction. However, with some chronic conditions, snapping hamstrings, and friction syndromes, dynamic ultrasonography may be useful to better evaluate the patient.^11^ Although most distal hamstring injuries will not be found on radiographs, it is important to evaluate the injuries with radiography to ensure that there is not a bony avulsion of the fibular head.

## Acute Injuries to the Distal Hamstrings

### Acute Lateral Side Distal Hamstring Injury

#### Epidemiology and Presentation

There is essentially only one acute lateral sided hamstring injury that can occur, which is a biceps femoris strain or avulsion, although other nonhamstring lateral sided injuries such as fibular collateral ligament sprain and popliteus tendon strain remain in the differential. As mentioned in the last section, distal biceps femoris injuries are a common component of multiligamentous and posterolateral corner knee injuries. Although it is relatively rare, an isolated distal biceps femoris tendon injury can have a profound effect on an athlete's ability to participate in sport, due to the biceps femoris's contributions to knee flexion strength, as well as dynamic and static stability of the lateral knee. Acute injuries to the distal biceps femoris in athletes occur mainly from noncontact injuries with knee hyperextension and hip flexion.^5^ The most commonly reported mechanism in the literature is within soccer athletes while kicking the ball with simultaneous knee hyperextension and hip flexion, with sprinting in track athletes being the second most commonly reported mechanism.^5^ The knee passively being forced into simultaneous hyperextension, and valgus has also been reported as a mechanism of injury.^5^

#### Examination and Imaging

Athletes sustaining a distal biceps femoris avulsion can present with pain around the posterolateral knee, ecchymosis, and/or reported weakness of the hamstrings compared with the contralateral side. On clinical examination, the provider should assess resisted knee flexion from the prone position, looking for either pain or weakness with resisted use. In addition, palpation can be notable for a defect or gap just proximal to the fibular head in the case of a complete tear or avulsion. The uninjured contralateral leg serves as a good comparison for both palpation and resisted strength testing. When an injury to the lateral side of the knee has occurred, a good neurologic examination should be done as the common peroneal nerve may also sustain a traction injury. Because of the high incidence of biceps femoris avulsions with multligamentous knee injuries, a complete ligamentous examination should be done. If a multiligamentous knee injury is suspected, we would recommend obtaining ankle brachial index measurements as part of the vascular examination.

**Figure 1:**
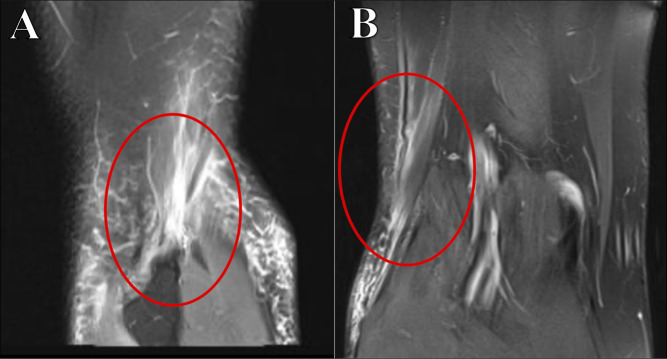
Sagittal T2 PDW MRI slice (**A**) demonstrating a distal avulsion of the biceps femoris tendon with the fibular head and retracted tendon end circled in red. There is fluid/edema between the visible tendon end and the fibular head with some tendon stump remaining on the fibular head. Coronal T2 PDW MRI slice (**B**) demonstrating retracted biceps femoris tendon. Note the nonlinear appearance or buckling of the tendon farther proximal to the ruptured end.

The definitive diagnosis of distal lateral biceps femoris tear is made with MRI, although ultrasonography can also be used in achieving the diagnosis (Figure 1). Because of the relative rarity of isolated biceps femoris injuries, we would recommend a knee MRI when a complete rupture is suspected over an ultrasonography to provide more detailed information about the biceps femoris tendon, including retraction, and to be able to detect any other concomitant acute knee injuries. Radiographs are valuable to assess the fibular head and ensure that the injury was not a bony avulsion.

#### Treatment

Low-grade strains and partial tears of the biceps femoris without retraction can be treated nonsurgically with a period of rest followed by physical therapy.^26^ By contrast, acute distal biceps femoris tendon avulsion injuries and high-grade partial or complete injuries at the musculotendinous junction should be treated surgically.^5^ For distal myotendinous junction injuries of the biceps femoris, Kayani et al.^27^ found that nonsurgical treatment had a 54% recurrence risk within 2 years of the injury, whereas surgical repair was able to achieve 100% patient satisfaction and return to preinjury sport level with no recurrence in 34 athletes. Similarly, Thompson et al published their results of acute surgical repair of distal biceps femoris avulsion injuries in professional athletes using suture anchors^8^; this study included 22 professional athletes who underwent surgical repair with all able to return to their previous level of sport around 16 weeks ( ±8.7 weeks). None of their patients had a recurrence at the 2-year mark, and none sustained a major complication from surgery.

**Figure 2:**
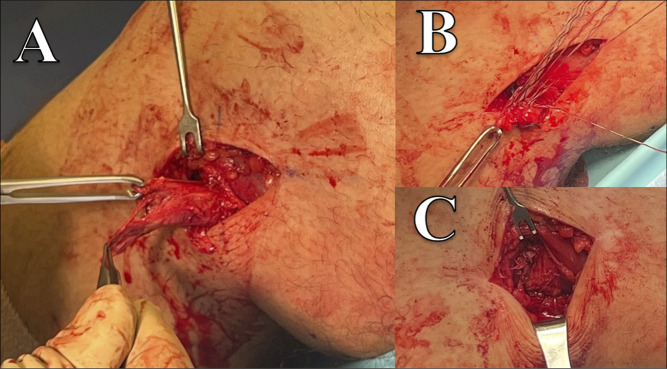
Clinical photographs demonstrating isolated complete distal avulsion of biceps femoris tendon from the fibular head (**A**) and repair of the tendon back to the fibular head. Photograph (**B**) demonstrating placement of repair stitches through the biceps femoris tendon after anchor placement into the fibular head, and photograph (**C**) demonstrating completed repair after sutures were tied.

To perform repair of a distal biceps femoris avulsion, the senior author uses a curvilinear incision with length based on level of injury or amount of retraction. For a mildly retracted bony avulsion injury, a 4- to 6-cm incision may be all that is required; however, for severe retraction or injuries extending to the musculotendinous junction, the incision may approach 10 cm. The incision runs from the level of the fibular head/neck up toward the lateral epicondyle of the femur, usually traveling just posterior to the lateral epicondyle. One should be aware that with complete avulsions of the biceps femoris, the common peroneal nerve may be displaced from its normal anatomic location. The senior author routinely locates the common peroneal nerve and performs a neurolysis of the nerve when repairing a biceps femoris avulsion. If the injury is a complete rupture at the musculotendinous junction, repair may be done with sutures using Kessler-style stitches for end-to-end repair. If a distal avulsion injury has occurred, suture anchors may be used in the fibular head. The senior author's preference is to use two, small, double-loaded, all-suture anchors placed into the fibular head, allowing for four total repair stitches (Figure 2). Having two separate anchors allows for better recreation of the anatomic biceps femoris footprint, which is broad. After repair, the knee may be allowed passive motion, but motion should be restricted to prevent full extension with some suggesting a limit of 30 and others 60° short of full extension. The senior author typically begins working on full extension after 2 weeks postoperatively in an attempt to avoid development of loss of terminal extension. Patients should be advised against active hamstring use until approximately 6 weeks postoperatively.

It should be noted that similar to proximal chronic hamstring injuries undergoing surgical intervention, the surgical repair of a chronic distal avulsion of the biceps femoris has been shown to have greater duration of time until return to play, reported at greater than 9 months,^28,29^ and poorer outcomes than with acute repair.^30^ Also, delays in surgery are associated with increased scar formation, which can lead to tethering to adjacent neurological structures and cause paresthesia and/or motor weakness.^27^ We recommend surgical treatment of complete injuries of the distal biceps femoris within a two-week window from time of the injury when possible.

### Acute Medial Side Distal Hamstring Injury

#### Epidemiology and Presentation

The acute injury to the medial distal hamstrings that occurs most often is to the semitendinous tendon. Isolated rupture of the semitendinosus distally is overall rare, with one recent systematic review only finding 22 instances reported in the literature ^10^ whereas another recent systematic view reported 52 instances.^31^ Distal semimembranosus avulsion or musculotendinous junction injuries have also been reported but are even more rare than semitendinosus injuries.^6,31^ Injuries to the medial hamstrings appear to be more common in high-level athletes than the general population. Specifically, this injury tends to occur with sprinting,^9,10,31^ although hyperextension of the knee is another possible mechanism. When presenting, patients will often report hearing an audible pop and feeling concomitant sharp, searing pain. Patients may note a feeling of a lump in their distal posterior thigh, and may have pain with passive full knee extension.^9^ When the patient presents with these constellations of findings, an acute semitendinosus rupture should be suspected. From the most recent systematic review of acute isolated semitendinosus injuries, an interesting demographic finding was that all individuals were males. In addition, the breakdown in location of injury was found to be 78% of injuries involved complete avulsions from the medial tibia, and 22% of tears were localized to the musculotendinous junction.^10^

#### Examination and Imaging

**Figure 3:**
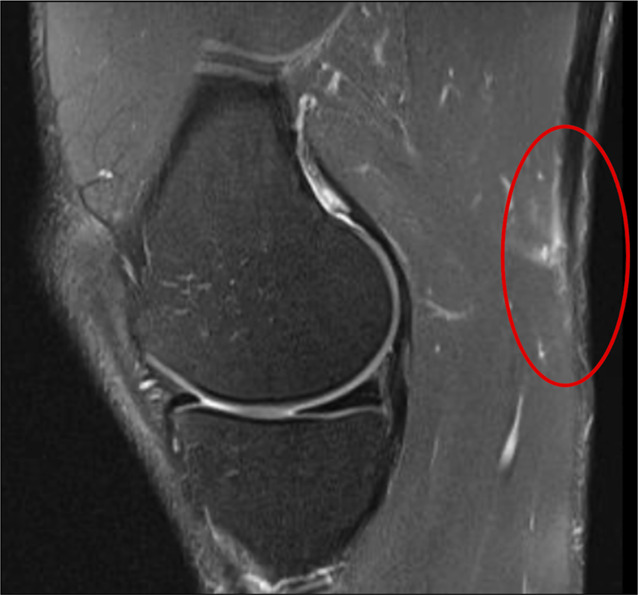
Sagittal T2 PDW MRI slice demonstrating a distal avulsion of the semitendinosus tendon with the tendon stump circled in red. The MRI was taken in the subacute phase such that there is minimal edema and/or fluid collection present.

Similar to when examining a patient with an acute lateral sided hamstring injury, a good ligamentous and neurovascular examination should be done for any patient with concern for an acute distal hamstring injury occurring medially. The hallmark finding of a ruptured and retracted semitendinosus tendon is a palpable posterior or posteromedial mass, which also tenders to palpation.^9^ In certain instances, particularly chronic ruptures, puckering of the skin of the popliteus fossa can be observed with active use of hamstring musculature.^9^ Strength testing can demonstrate weakness in active knee flexion. MRI can be used to confirm diagnosis with high accuracy for this diagnosis (Figure 3). Ultrasonography is another option for imaging of this injury but is subject to variability in effectiveness dependent on examiner.

#### Treatment

In an athlete, a complete distal tendon rupture is usually a surgical indication. Similar to avulsions of the biceps femoris, nonsurgical management of medial hamstring avulsions carries a high risk of failure to return to preinjury activity.^9,27,31,32^ In terms of treatment for an isolated acute semitendinosus rupture, it has been shown that 42% to 53% of patients initially treated nonsurgical failed and went on to require surgical intervention.^9,31^ Furthermore, when nonsurgical treatment failed and patients were converted to surgical treatment, the mean time to return to sport was extended by approximately 3 months, from 1.0 to 4.3 months in a case series in which excision of the tendon stump was done and from 4.2 to 7.6 months in a systematic review where 74.5% of patients undergoing surgery received either repair or tenodesis.^9,31^

**Figure 4:**
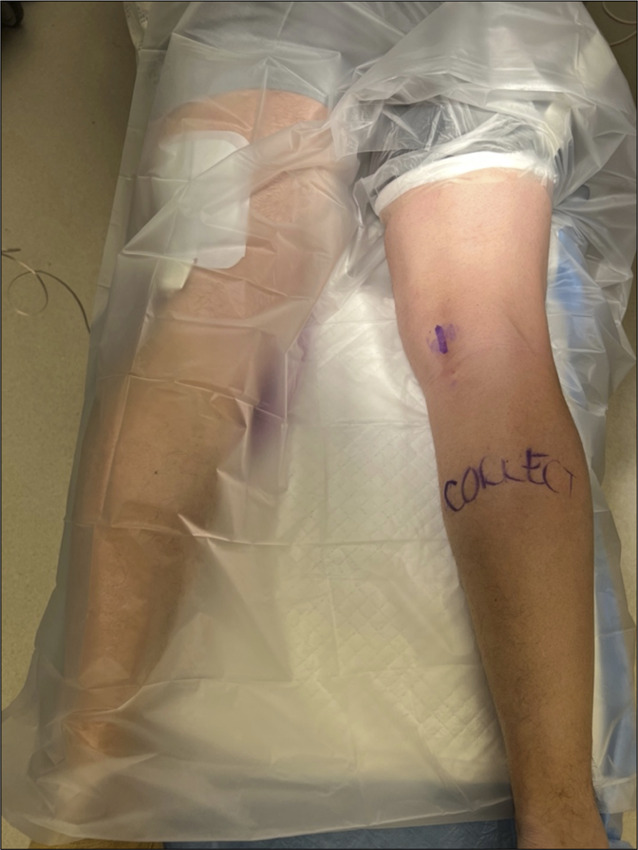
Clinical photographs demonstrating marked incision of semitendinosus stump after distal avulsion with incision centered over palpable stump.

The surgical options for a distal avulsion of the semitendinosus are (1) direct repair with suture anchor, (2) débridement of the tendon stump with tenodesis to the semimembranosus, and (3) open excision of the avulsed semitendinous stump without tenodesis.^6,9,29,31,33^ Direct repair to the proximal tibial footprint can be done using supine positioning with an incision over the anteriomedial proximal tibia in similar location to an incision for a medial hamstring harvest. In the largest case series detailing this surgical options, excellent outcomes were achieved in 93% of the 14 patients in the series with an average return to sport at 5.5 months after surgery.^29^ Tendon débridement and tenodesis to the adjacent semimembranosus can be done through a prone incision directly over the avulsed stump of the semitendinosus (Figure 4).^8^ If the stump is not palpable, measuring on the MRI can help guide placement of the incision. The hamstring fascia is incised, and the semitendinosus stump is identified, while protecting the saphenous nerve. The semitendinosus is then sutured to the adjacent semimembranosus tendon under low to moderate tension. Thompson et al^33^ reported good results with this technique; however, they had only a small cohort that was heterogenous, including acute semitendinosus injuries, chronic semitendinosus injuries, and patients with previous semitendinosus harvest for ACL reconstruction who presented with chronic pain, weakness, or feelings of instability. The other primary method of surgical treatment is complete resection of the distal semitendinosus tendon with care taken to remove all inflamed and fibrotic peritenon and to free up all adhesions.^9^ The same incision and approach as is used for semitendinosus tenodesis is used. Cooper et al did find that the tendon was usually inflamed and markedly enlarged, often preventing the use of normal hamstring harvesters during débridement.^9^ Postoperative protocols were fairly similar between different surgical treatments, and on average, overall return to sport time was 2.5 months (±1.4 months); Of note, return to play was markedly shorter in athletes with avulsion injuries (2.2 months) compared with those with tears from the musculotendinous junction (3.8 months).^10^ Most studies show high postoperative satisfaction and ability to return to sport after surgery, regardless of surgery type, although no comparative studies analyzing differences in outcome based on the type of surgery exist.^6,8–10^

As distal semimembranous injuries are exceedingly rare within the literature, there is scant evidence to guide surgical treatment. Lempanien et al describe the largest series of distal semimembranosus injuries, which included four partial and one complete tear at the distal musculotendinous junction. Although the one complete tear underwent direct surgical repair with suture and was able to return to sport at preinjury level at 6 months, the four partial injuries underwent delayed repair with initial trial of nonsurgical treatment. Despite surgical intervention, none of the four athletes were able to return to sport at preinjury level, and all were classified as having a poor to fair outcome. Poorer outcomes with nonsurgical or delayed treatment with semimembranosus distal injuries may be due to the notable function of the semibranosus in contributing to knee stability as part of the posteromedial corner complex.^34^ In a recent case report, Sonnery-Cottet reported excellent outcome and return to professional rugby at prior level for an athlete who suffered a complete avulsion of the semembranosus and underwent anchor repair to the proximal tibial insertion.^34^

## Chronic Pathologies of the Distal Hamstrings

There are three chronic conditions of the distal hamstrings that will be discussed, which will be further subdivided into medial or lateral injuries. The medial two conditions are pes anserine bursitis and medial snapping hamstring, and the lateral condition, lateral snapping hamstring, will all be discussed in further detail below.

### Chronic Medial Distal Hamstring Pathologies

The pes anserine is the confluence of the sartorius, gracilis, and semitendinosus tendons at their attachment site on the proximal, medial tibia. A small bursa is located between the tendons and the medial tibial to help control friction as the tendons glide over the medial tibia. Mechanical derangement, direct trauma, obesity, and overuse have all been implicated in developing pes anserine bursitis; it is also more commonly found in females than males and in patients who already have evidence of knee osteoarthritis.^35–37^ Regardless of the mechanism, inflammation of this bursa or the tendons can lead to pes anserine bursitis. Patients with this condition will typically present with medial sided knee pain, which will be provoked by climbing or descending stairs, moving from a seated to standing position, and other activities requiring active knee flexion.^38^ Often, patients will describe weakness and decreased range of motion with knee flexion. One of the key findings is tenderness over the insertion of the pes anserine tendons. A described physical examination maneuver for detecting pes anserinus bursitis involves testing resisted knee flexion while the leg is positioned in external rotation, with reproduction of pain denoting a positive test.^39^ The diagnosis of pes anserine bursitis is largely a clinical diagnosis, with the combination of the above findings. Imaging studies, such as radiograph, ultrasonography, and MRI typically are not used to definitively diagnose this condition. However, they can be helpful in ruling out other conditions for cases that are unclear.^38^ In addition, it is important that the treating provider remember that the differential diagnosis for pes anserine bursitis can include infection to the area, gout, medial meniscus pathology, L3/L4 radiculopathy, medial collateral ligament injuries, and various cysts, ganglions, or masses around the knee. Treatment for pes anserine bursitis can include rest, ice, NSAIDs, weight loss, physical therapy, and steroid injection.^40,41^ Physical therapy protocols emphasize strengthening of adductor and quadriceps muscle groups, particularly in the terminal 30° of extension, in addition to stretching of the pes anserine tendons.^39^ Surgical intervention is usually not needed for treatment, but bursa irrigation and drainage, bursal excision, and treatment of concomitant or inciting pathology are described in the literature.

**Figure 5:**
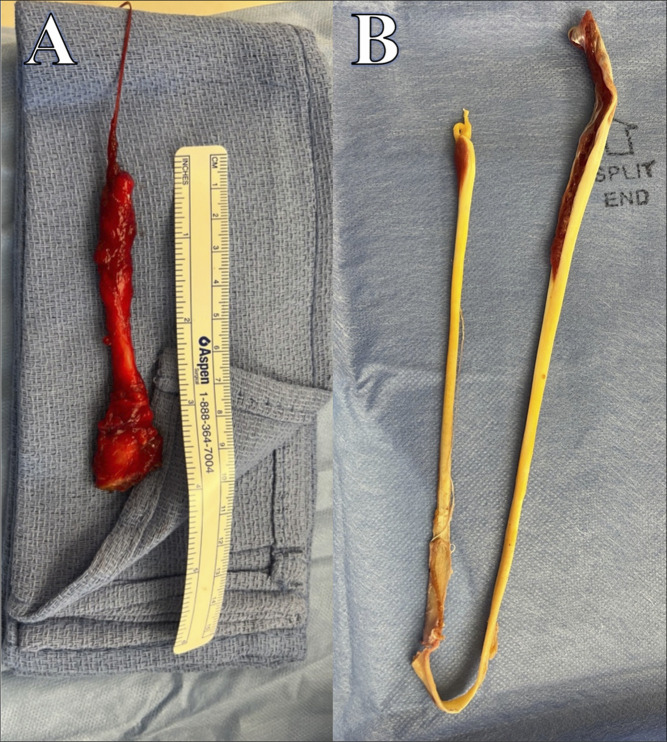
Clinical photographs demonstrating (**A**) excised semitendinosus tendon stump extending just into musculotendinous junction after an isolated distal avulsion of the semitendinosus tendon. Tendon stump excised through open posterior approach (**B**) excised semitendinosus (right) and gracilis (left) tendons for snapping medial hamstring syndrome. Tendons excised using an open hamstring harvester through a small anterior incision over the pes insertion on the proximal tibia.

The other chronic medial condition of the distal hamstring is snapping of the medial hamstring tendons. This is an uncommon condition and is not well described in the literature. Because of the paucity of data, clinical knowledge of the condition can be lacking, and this may make it hard to diagnose and even lead to unnecessary surgical procedures. Medial snapping hamstring occurs when the pes tendons subluxate or “snap” over the proximal medial tibial plateau or other soft-tissue structures. Patient's will present with pain over the posteromedial knee and an audible or palpable popping on examination with flexion and extension of the involved knee (Video 1).^42^ Imaging modalities are typically secondary to clinical evaluation in terms of making the diagnosis. However, dynamic ultrasonography can be helpful to determine if there is an underlying cause for the snapping and to elucidate which specific structures appear to be involved. The etiology is unknown, but LaPrade et al documented scarred/adhered bursa in all cases.^12^ Nonsurgical treatment can be trialed with physical therapy; however, most cases ultimately require surgical intervention. One small case series treated one patient with open hamstring harvest with a hamstring harvester; two other patients were treated with release of the tendons off the insertion of the tibia. All patients had a return to normal knee function with no further catching episodes at a minimum of 2-year follow-up.^13^ An open posterior approach can also be used to perform a débridement, excision, or tenotomy of the involved tendon as an alternative option (Figure 5).

### Chronic Lateral Disorder of the Distal Hamstring

The last portion of this review will discuss more chronic or overuse injuries to the lateral side of the knee. Although overuse pathologies of the lateral knee such as iliotibial band friction syndrome are common, overuse pathologies involving the biceps femoris are uncommon. The two chronic or overuse pathologies of the biceps femoris are biceps femoris tendinopathy and snapping biceps syndrome.

Biceps femoris tendinopathy has been reported in young athletes, although only 12 total cases are described in the literature.^43,44^ However, it is possible that these injuries are underreported and potentially mistaken for other conditions of the lateral knee.^43^ This problem should be managed conservatively with rest, anti-inflammatories, and physical therapy, as there is usually success in resolving symptoms with this management. When conservative care is unsuccessful, surgical treatment with débridement of poor-appearing tendon and repair with or without suture anchors into the fibular head have been reported to result in excellent outcomes.^43^

Snapping distal biceps femoris syndrome represents a pathologic condition in which the distal biceps femoris tendon snaps over the fibular head, whereas the knee is going through range of motion. Patients may present with complaints of lateral sided knee pain, snapping around the knee, or feelings of instability.^11^ One rare cause of this condition is fibular head hypertrophy.^45,46^ However, the more common cause is an anatomical insertional difference of the tendon usually either too anterior on the fibular head or mostly on the tibia.^11^ The recognition of this anatomic variation has led to the discovery of patient presenting with what has been described as a friction syndrome of the distal biceps femoris tendon, which can have similar symptoms without the audible or visible snapping.^11^ Of note, this distal biceps femoris attachment friction syndrome does appear to be more common in cyclists. Catonne et al^11^ describe the four types of biceps femoris distal insertion and discussed their treatment algorithm for patient presenting with this issue. In addition to detailed history and physical examination, an MRI can be useful to rule out other pathology, although the optimal imaging option is dynamic ultrasonography, which can confirm the diagnosis. For treatment of snapping biceps femoris, surgical management has proven to be very effective. In terms of technique, surgery involves removing the tendon and reattaching to the posterolateral aspect of the fibular head.^47^ This is done through an open posterolateral approach, as described for repair of biceps femoris avulsion with consideration for common peroneal nerve neurolysis as well, particularly if anchors will be placed into the fibular head for fixaton.

## Conclusion

Hamstring injuries are one of the most common injuries sustained by athletic populations. Although most these are proximal hamstring injuries, distal injuries can occur too. It is our hope that this article may expand the reader's knowledge on distal hamstring anatomy and pathology, and aid with diagnosis and treatment. It has been shown that these injuries can be sustained by elite athletes, and that this subset of the population may have difficulty returning to their previous level of activity with nonsurgical treatment. In addition, patient experiencing chronic symptoms may be misdiagnosed and even undergo improper surgeries if the treating physician is unfamiliar with diagnosis.
